# Using Resonance Frequency Analysis to Compare Delayed and Immediate Progressive Loading for Implants Placed in the Posterior Maxilla: A Pilot Study

**DOI:** 10.2174/1745017901814010801

**Published:** 2018-10-18

**Authors:** Mohammed Jasim AL Juboori, Mohammed Ahmed AL Attas, Raquel Zita Gomes, Basma Fathi Alanbari

**Affiliations:** 1 Department of Periodontology, Al-Rafidain College, Baghdad, Iraq; 2 Department of Prosthodontic and Dental Implant, Riyadh Dental Center, King Saud Medical City, Riyadh, Saudi Arabia; 3 Department of Oral Surgery, Faculty of Dental Medicine, University of Porto, Porto, Portugal

**Keywords:** Maxillary bone, Provisional crown, Implant stability, Bone density, Bone-Implant Contact (BIC), Resonance Frequency Analysis (RFA)

## Abstract

**Objective:**

Implants placed in the posterior region of the maxilla have a high incidence of implant failure due to poor bone quality, especially when immediate implant loading is needed. Immediate Progressive Loading (IPL) can enhance bone quality and may offer an alternative solution when Immediate Implant loading is needed.

**Methods:**

Six patients (one male and five females; 34-62 years old) were included in this study. Twelve implants were inserted in the posterior region of the maxilla. Resonance Frequency Analysis (RFA) was performed at the time of implant placement and after 1, 2, 3 and 6 months. ISQ (Implant Stability Quotient) values were compared between the Delayed Loading (DL) group after 2 months and the Progressive Loading (PL) group and between different time points for each group.

**Results:**

At implant placement, the mean ISQ values for PL and DL implants were 63 and 57, respectively. One month after implant placement, the mean ISQ value for PL implants was 73.

Two months after implant placement, the mean ISQ value for PL implants was 75. Three months after implant placement, the mean ISQ values for PL and DL implants were 76 and 69, respectively. Six months after implant placement, the mean ISQ values for PL and DL implants were 79 and 76, respectively.

**Conclusion:**

Despite its limitations, this pilot study indicated that compared to DL, PL can enhance bone density and implant stability, resulting in greater early functionality and fewer surgery sessions.

## 1 INTRODUCTION

Bone density is variable from the upper jaw to the lower jaw and from the anterior to the posterior area of the jaws. According to the Lekholm and Zarb classification [1], the posterior region of the maxilla has the lowest bone density. This area is characterized by a thin layer of cortical bone enclosing a core of low-density trabecular bone. Bone density plays a crucial role in primary and secondary implant stability and the implant success rate [2-4]. Generally, a greater incidence of implant failure occurs in soft bone, as well as in the posterior maxillary area [5-9]. Some special considerations must be taken into account for implants placed in the posterior maxillary area; long, threaded, surface-treated implants with a wide diameter are usually considered the best option [3, 10-13].

Implant placement under size socket preparation is preferable to obtain greater primary implant stability. Subcrestal implant placement can engage more cortical bone and decrease stress on the surrounding bone [14, 15].

Progressive implant loading in soft bone was invented by Misch CE., to increase the density of soft bone after implant placement [16].

Misch CE., [16] Progressive Loading (PL) allows the bone to mature during the loading period without overloading the implant. Ban *et al*., [17] compared 2 groups of patients who underwent Immediate Progressive Loading (IPL) with a group of patients who underwent Delayed Loading (DL). A significant increase in the Bone-Implant Contact (BIC) and a decrease in the vertical bone loss were reported in the IPL group. In this case report, we describe the use of noninvasive tools to measure implant stability. We think that follow-up with patients is crucial to ensure no disruption of bone osseointegration [18]. Resonance Frequency Analysis (RFA) is considered an accurate method that can be used to measure implant stability in a resonance-dependent manner [18-23]. Most previous studies have not included a long follow-up period when comparing different implant loading techniques and these studies usually end after delivery of the prosthetic part. In our study, we followed up on implant stability for 6 months after loading, and we compared the results obtained with DL and IPL using RFA.

## 2 MATERIALS AND METHODS

The current randomized controlled clinical trial compared the outcomes of two implant loading techniques in a split-mouth design. One implant was placed in one quadrant of the jaw (maxilla) as part of the trial group, PL, while another implant was placed in a similar location in the contralateral jaw quadrant as part of the control group, DL, during the same surgical appointment.

The inclusion criteria were as follows:

1. Bilateral posterior missing teeth in the upper jaw.
2. Sufficient attached gingival keratinized tissue to support an adequate attached tissue cuff around the implant.
3. Adequate height space.
4. Normal occlusion with anterior and canine guidance.

The exclusion criteria were as follows:

1. A narrow alveolar ridge that could not provide 2 mm of bone around the implant.
2. An extracted socket that had been healing for less than 4 months.
3. Alveolar bone grafting or maxillary sinus lifting.
4. Patients with para-functional habits.
5. Debilitated patients.

All performed procedures were explained verbally and in writing to the patients, and all patients signed an informed consent form for the implant placement procedure. Follow-up for data collection was scheduled for 24 weeks after implant placement.

Orthopantomogram (OPG) X-ray images were also obtained to evaluate the vertical bone height and vital anatomical structures. The implant design was tapered, and the surface was modified (Sandblasted, Large-grit, and Acid-etched, SLA) and threaded up to the top (Superline, Dentium). Implants were placed manually into their final positions using a ratchet with an insertion torque of 20-35 Ncm. The implants chosen for PL required a torque of more than 30 Ncm, while the implants that achieved less than 30 Ncm were left as submerged or DL implants.

**Fig. (1):**
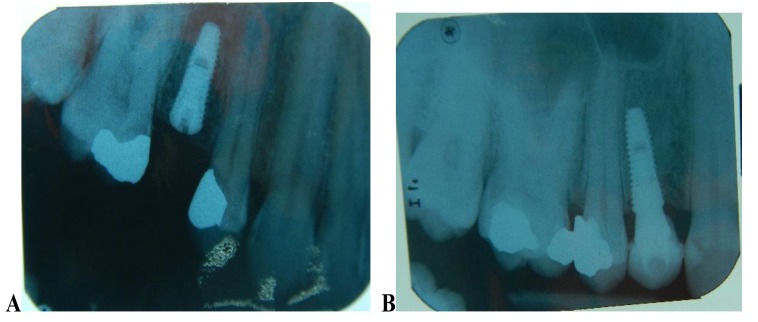
Postoperative radiograph shows the split-mouth technique when choosing delayed implant loading (submerged) on the right side in the second premolar (**A**), while immediate progressive loading was performed for the same patient on the left side with temporary abutment and a crown fabricated from composite (**B**).

**Fig. (2):**
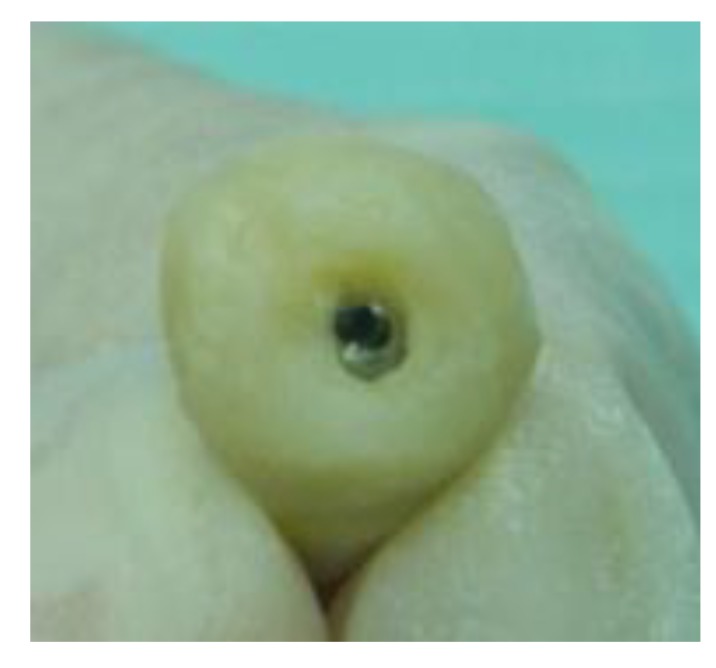
Using temporary abutment and composite material to contour the crown during the first 3 months. The crown increased in diameter every month and was out of occlusion for the first two months.

**Fig. (3):**
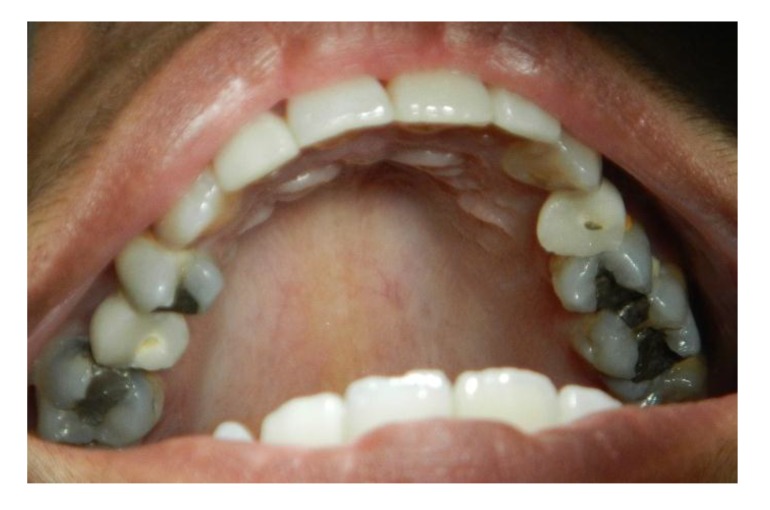
Acrylic provisional crowns placed on the permanent abutment for both implants and were lifted for 3 months. The type of contact with the crowns was reported only in centric occlusion.

**Fig. (4):**
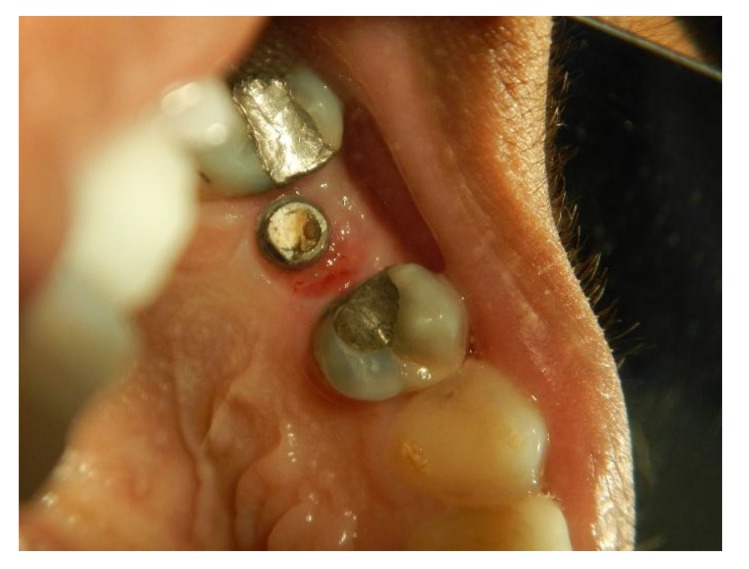
Gingival emergence profile created with temporary composite and provisional acrylic crowns. Last RFA measurement was performed before an impression was taken on the abutment level.

**Fig. (5):**
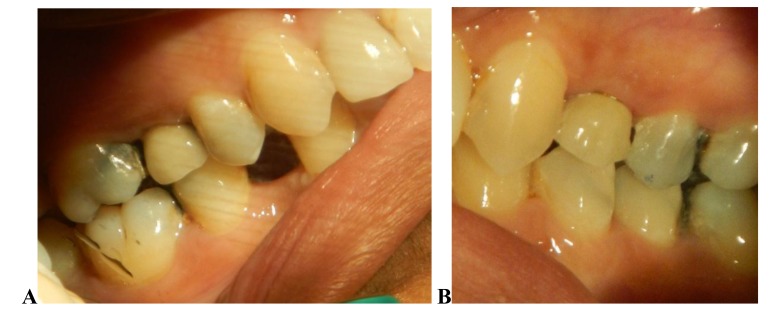
Porcelain fused to metal crown as a definite crown issued for both implants (A, delay loaded implant and B, immediate loaded implant) after 6 months of implant placement.

The implants were placed subcrestally at approximately 1 mm. RFA measurements were obtained for both implants at the time of surgery. A cover screw was placed for DL implants and was covered with gingival tissue; primary closure was performed using the submerged technique. PL implants were immediately loaded using a temporary plastic abutment, and crowns were fabricated from light-cured composite material. At this stage, the crowns were out of occlusion with a narrow occlusal table and no adjacent contact. The flaps were adapted to the temporary crowns, and the gingival tissue was sutured around them. Periapical radiographs were postoperatively obtained for both implants and considered basic radiographs (Fig. **1**). Postoperative instructions were given to patients. After 10 days, patients returned for suture removal and examination. Both implant sites healed uneventfully, and suture removal was performed. One month after implant placement, the temporary abutments and crowns were unscrewed. RFA measurements were obtained. The crowns were modified by increasing the width of the occlusal table, and the crowns were in mesial contact with an adjacent tooth (upper right first molar). At this stage, the crowns were out of occlusion (no increase in crown height). The crowns were polished and screwed into the fixtures again (Fig. **2**). Two months after implant placement, the patients returned again for the third stage of crown modification. The temporary abutment and crown were unscrewed. The crowns were modified by increasing their height, and the crowns were in occlusal contact with an agonist only in the axial direction. There was no occlusal contact during excursion movement, and only central contact remained. Three months after implant placement, the temporary abutments and crowns were unscrewed in the PL group, and implants in the DL group were exposed after 3 months. RFA measurements were obtained in both groups. Healing abutments were placed and left in place for one month for soft tissue maturation. Four months after implant placement, impressions were taken in both groups. The closed tray impression technique with hex impression coping was performed. Bite registration was conducted using a wax bit rim, and the jaw relationship was measured using a laboratory-created titanium hex straight abutment and an acrylic provisional crown. The abutments were screwed in and tightened to 30 Ncm for both groups of implants, and the provisional crowns were cemented using temporary cementation (Fig. **3**). Occlusion was assessed and adjusted. Only centric occlusion was allowed, and there was no contact during the excursion movement. Periapical radiographs were obtained for both implants and considered basic prosthetic radiographs for further follow-up after implant loading. After 6 months, the provisional crowns were removed (Fig. **4**), and the abutments were unscrewed and removed. RFA measurements were obtained for both groups of implants. The abutments were screwed in again, and direct impressions were taken (at the abutment level). Porcelain fused to metal was fabricated and issued as a permanent crown after provisional crown removal (Fig. **5**).

## 3 RESULTS

Six patients (one male and five females; 34-62 years old) were included in this study. A total of 12 implants were inserted in the posterior region of the maxilla. Each patient received two implants in a split-mouth design. Implants were distributed between molars and premolars (Table **1**). Neither IPL nor DL implants demonstrated rotational movement during cover screw removal or tightening for Smartpeg placement, indicating good implant stability.

All patients completed the 6-month follow-up examination. Postsurgical wound healing was uneventful in all cases, and none of the cases were complicated by continuous pain, limited mobility, radiographic radiolucency or infection. The cumulative success rate was 100%.

### 3.1 RFA Measurements at Implant Placement

The mean Implant Stability Quotient (ISQ) values for PL and DL implants were 63 and 57, respectively (Table **2**); these values were significantly different.

### 3.2 RFA Measurements One Month after Implant Placement

The mean ISQ value for PL implants was 73.

### 3.3 RFA Measurements 2 Months after Implant Placement

The mean ISQ value for PL implants was 75.

### 3.4 RFA Measurements 3 Months after Implant Placement

The mean ISQ values for PL and DL implants were 76 and 69, respectively. After 3 months, the ISQ values for PL and DL implants increased by 13 and 12, respectively.

### 3.5 RFA Measurements 6 Months after Implant Placement

The mean ISQ values for PL and DL implants were 79 and 76, respectively.

## 4 DISCUSSION

With this study, we aimed to compare DL implants (submerged implants) and IPL implants after exposure to a gradual increase in occlusion stress on the implant until contact with the agonist was achieved. In our study, we avoided occlusal contact for IPL implants during the 2-month healing period, and we allowed gradual contact with the crown. Our procedure mimics “tooth eruption”, which may exert some stress on the surrounding bone while simultaneously allowing the bone to mature [16]. No contact occurred during excursion at any stage (immediate composite, acrylic provisional and permanent crowns) to prevent lateral or angled force on the implant.

According to numerous studies, immediate loading should start with nonfunctional loading [24, 25], as immediate functional loading might cause micromovement, leading to fibrous tissue formation instead of bone formation around the implant [26-29].

Moreover, immediate placement of implants with overloading or loading with more than 150 µm can cause implant micromovement and soft tissue formation [30-32].

In our study, the primary stability of immediately loaded implants was above 60 ISQ, while most of the delayed implants showed low stability (as low as 20 ISQ). Ostman and colleagues [33] used a primary stability value of 60 ISQ as a threshold value for immediate loading. Another study found an increase in implant loss with ISQ values lower than 60 for immediately loaded implants [34]. Primary stability is crucial for the achievement of implant secondary stability, as implants with good primary stability have a shorter healing period with an increase in stability during the healing period [35, 36].

During the follow-up period, the stability of IPL implants increased by 10 ISQ after one month, while the increase was only 2 ISQ in the second month. This result indicates that implant stability increases to a greater extent during the first month of healing. When we compared IPL and DL implants after 3 months, we found that the increase in the ISQ was the same (13 ISQ for IPL implants and 12 ISQ for DL implants). After 6 months of loading with a provisional crown, the IPL implants continued to show an increase in stability, although the increase was minimal (only 3 ISQ). Conversely, DL implants showed a larger increase of 8 ISQ. At the 6-month reading, both techniques achieved nearly the same implant stability but over different time periods.

Immediately loaded implants may show a decrease in stability after one month [37]; however, we did not observe this phenomenon in our study when implants were progressively loaded. Compared with DL implants, immediately loaded implants can recover their stability and show increased stability after completion of the healing period [37]. Many studies have reported that implant loading can increase implant stability and bone density of the bone around the implant, especially in soft bone [34, 38].

Makowiecki *et al*., [39] concluded that there was no significant difference between early loaded implants and DL implants after 6 months. In that study, the authors used functional loading for early implants after 6 weeks; for DL implants, loading was delayed until 15 weeks. If we compare our results to those of Makowiecki *et al*. [39], our implant loading timing was different, but we obtained the same implant stability after 6 months. This result may be due to the maturation of the bone and formation of lamellar bone at this time point. Bone maturation may have occurred earlier in the immediate or early loaded implants and stopped at the same bone maturation level observed in the DL implants.

Another factor that may contribute to the success of immediate implant loading is bone nature and volume, as our implants were placed in native bone with sufficient height and width for regular implants (4.0 mm x 10 mm) [40]. Primary implant stability is compromised when the implant is placed in an augmented ridge (either vertical or horizontal bone grafting) [41].

In our study, the implant crowns in the PL group were composed of many types of crown materials, including composite, acrylic and porcelain fused to metal. The type of crown material has some influence on the force transmitted to the implant [40]; for example, when composite material is exposed to excessive force, it does not adequately transmit the force to the implant itself and may become fractured.

Another advantage of PL is the ability to contour and shape the gingival tissue, creating an emergence profile for the permanent crown.

RFA is a noninvasive intraoral method that is designed to reflect the bone/implant interface. This method is useful for documenting clinical implant stability during the healing period and throughout routine follow-up [17, 11]. The drawback of this method is that the abutment must be disconnected from the implant to allow the transducer to be screwed in during the follow-up period. This issue may cause disruption of the biological seal between the gingival collar and abutment. After assessing the results of this study, we would like to offer some surgical suggestions that could help to achieve better primary implant stability (Table **3**). Prosthetic parts also deserve special consideration as factors related to these parts may affect the prognosis of patients with immediately loaded implants (Table **4**).

## Figures and Tables

**Table 1:** Implant placement positions among the patients.

**Type of Loading**	**1^st^ Premolar**	**2^nd^ Premolar**	**1^st^ Molar**	**2^nd^ Molar**	
**Immediate**	**1**	**3**	**1**	**1**	
**Delayed**	**1**	**2**	**2**	**1**	

**Table 2:** Mean RFA measurement during the six-month healing period.

**Time**	**Immediate Loading**	**Delayed Loading**
**At surgery**	**63**	**57**
**1^st^ month**	**73**	**----------**
**2^nd^ month**	**75**	**----------**
**3^rd^ month**	**76**	**69**
**6^th^ month**	**79**	**76**

**Table 3:** Surgical considerations for progressive loading implant.

**No.**	**Factors**
1- 2- 3- 4- 5-	Narrow diameter implant avoided (less than 4 mm) Subcrestal implant placement Aggressive type of implant Implant surface treated Under size socket preparation

**Table 4:** Prosthetic considerations for progressive loading implant.

**No.**	**Factors**
1- 2- 3- 4- 5-	Contraindicated for bruxism patients No angle implant or abutment Immediate loading is contraindicated with RFA less than 65 ISQ Crown out of occlusion on the first month One-month interval between abutment disconnection

## CONCLUSION

Although somewhat limited, this pilot study showed no significant difference between DL and IPL implants if all regulations are followed. Immediate loading was allowed for soft and hard tissue maturation with increased implant stability.
